# Diagnostic Criteria for Identifying Individuals at High Risk of Progression From Mild or Moderate to Severe Alcohol Use Disorder

**DOI:** 10.1001/jamanetworkopen.2023.37192

**Published:** 2023-10-10

**Authors:** Alex P. Miller, Sally I-Chun Kuo, Emma C. Johnson, Rebecca Tillman, Sarah J. Brislin, Danielle M. Dick, Chella Kamarajan, Sivan Kinreich, John Kramer, Vivia V. McCutcheon, Martin H. Plawecki, Bernice Porjesz, Marc A. Schuckit, Jessica E. Salvatore, Howard J. Edenberg, Kathleen K. Bucholz, Jaquelyn L. Meyers, Arpana Agrawal

**Affiliations:** 1 Department of Psychiatry, Washington University School of Medicine, St Louis, Missouri; 2 Department of Psychiatry, Rutgers Robert Wood Johnson Medical School, Piscataway, New Jersey; 3 Department of Psychiatry and Behavioral Sciences, State University of New York Health Sciences University, Brooklyn; 4 Department of Psychiatry, University of Iowa, Iowa City; 5 Department of Psychiatry, Indiana University, Indianapolis; 6 Department of Psychiatry, University of California San Diego Medical School, San Diego; 7 Department of Biochemistry and Molecular Biology, Indiana University, Indianapolis

## Abstract

**Importance:**

Current *Diagnostic and Statistical Manual of Mental Disorders* (Fifth Edition) (*DSM-5*) diagnoses of substance use disorders rely on criterion count–based approaches, disregarding severity grading indexed by individual criteria.

**Objective:**

To examine correlates of alcohol use disorder (AUD) across count-based severity groups (ie, mild, moderate, mild-to-moderate, severe), identify specific diagnostic criteria indicative of greater severity, and evaluate whether specific criteria within mild-to-moderate AUD differentiate across relevant correlates and manifest in greater hazards of severe AUD development.

**Design, Setting, and Participants:**

This cohort study involved 2 cohorts from the family-based Collaborative Study on the Genetics of Alcoholism (COGA) with 7 sites across the United States: cross-sectional (assessed 1991-2005) and longitudinal (assessed 2004-2019). Statistical analyses were conducted from December 2022 to June 2023.

**Main Outcomes and Measures:**

Sociodemographic, alcohol-related, psychiatric comorbidity, brain electroencephalography (EEG), and AUD polygenic score measures as correlates of *DSM-5* AUD levels (ie, mild, moderate, severe) and criterion severity–defined mild-to-moderate AUD diagnostic groups (ie, low-risk vs high-risk mild-to-moderate).

**Results:**

A total of 13 110 individuals from the cross-sectional COGA cohort (mean [SD] age, 37.8 [14.2] years) and 2818 individuals from the longitudinal COGA cohort (mean baseline [SD] age, 16.1 [3.2] years) were included. Associations with alcohol-related, psychiatric, EEG, and AUD polygenic score measures reinforced the role of increasing criterion counts as indexing severity. Yet within mild-to-moderate AUD (2-5 criteria), the presence of specific high-risk criteria (eg, withdrawal) identified a group reporting heavier drinking and greater psychiatric comorbidity even after accounting for criterion count differences. In longitudinal analyses, prior mild-to-moderate AUD characterized by endorsement of at least 1 high-risk criterion was associated with more accelerated progression to severe AUD (adjusted hazard ratio [aHR], 11.62; 95% CI, 7.54-17.92) compared with prior mild-to-moderate AUD without endorsement of high-risk criteria (aHR, 5.64; 95% CI, 3.28-9.70), independent of criterion count.

**Conclusions and Relevance:**

In this cohort study of a combined 15 928 individuals, findings suggested that simple count-based AUD diagnostic approaches to estimating severe AUD vulnerability, which ignore heterogeneity among criteria, may be improved by emphasizing specific high-risk criteria. Such emphasis may allow better focus on individuals at the greatest risk and improve understanding of the development of AUD.

## Key Points

**Question:**

Does emphasis on specific criteria for alcohol use disorder (AUD) improve the identification of individuals at risk for developing more severe AUD?

**Findings:**

In this cohort study, cross-sectional and longitudinal multimodal secondary analyses involving a combined 15 928 individuals indicated that endorsement of criteria empirically designated as representing greater severity of AUD was significantly associated with 2-fold increased likelihood of progression from mild-to-moderate AUD to severe AUD, even after accounting for total criterion count.

**Meaning:**

Emphasis on more severe criteria as indicators of vulnerability for severe AUD in current diagnostic approaches may increase detection of individuals with greater likelihood for disorder progression.

## Teaser

This cohort study examines data from the Collaborative Study on the Genetics of Alcoholism to assess whether specific diagnostic criteria for alcohol use disorder that are indicative of greater severity could help detect a greater likelihood of progression.

## Introduction

Alcohol use disorder (AUD), as defined by the *Diagnostic and Statistical Manual of Mental Disorders* (Fifth Edition)^1^ (*DSM-5*), is conceptualized as a syndrome of sustained problematic alcohol use and clinically significant impairment. A diagnosis is based on endorsement of 2 or more of 11 criteria assessing behavioral and physical manifestations of heavy alcohol use that occur in a 12-month period.^1^ Recent US estimates indicate that 11% and 30% of adults meet criteria for past-year and lifetime AUD, respectively.^2,3^ This level of disordered alcohol use results in significant social, economic, and public health costs.^4,5,6^

*DSM-5* AUD is diagnosed on a continuum of severity based on the number of criteria endorsed (2-3 = mild, 4-5 = moderate, ≥6 = severe),^1^ and identifying individuals at high risk for severe AUD is a priority. Several studies document increased comorbid burden and reduced likelihood of recovery as a function of increasing criterion count.^7,8,9^ However, count-based indices are limited by their equal weighting of criteria, suggesting all diagnostic criteria are interchangeable.^10^ Extensive cross-national psychometric evidence shows that certain AUD criteria (eg, withdrawal) are more likely to be endorsed by those with more severe AUD and may represent superior indicators of risk.^11,12,13,14^ Thus, criteria heterogeneity is an important factor to consider in the advancement of personalized treatment approaches.^15^ Whether “high-risk” AUD criteria endorsement results in differing associations with a wide range of psychiatric, genetic, and neurobiological correlates could further guide delineation of risk severity.

In light of the above evidence,^12^ here we investigate the impact of criteria heterogeneity within criterion count–based AUD diagnoses. As other research also suggests that severe AUD may be substantively different from mild or moderate AUD in terms of treatment needs,^16^ functional impairment,^17^ and other health sequelae,^18^ we sought to compare mild-to-moderate AUD (ie, endorsing 2-5 criteria, meeting criteria for either mild or moderate AUD) vs severe AUD while accounting for individual differences in criterion count among those with mild-to-moderate AUD. The current study aimed to (1) validate criterion count–based severity using *DSM-5* categorizations of AUD, including comparing mild-to-moderate vs severe AUD, in a sample of 13 110 individuals; (2) use item response theory (IRT) modeling to identify criteria indicative of greater severity; and (3) evaluate whether the presence of certain high-risk criteria, identified through IRT modeling, indexes greater hazards of developing severe AUD in a related cohort of 2818 adolescents and young adults.

## Methods

### Participants

The Collaborative Study on the Genetics of Alcoholism (COGA) is a family-based study with deep and repeated phenotyping of substance use disorders (SUDs), comorbid psychiatric disorders, and related traits (including polygenic liability and electrophysiological markers) designed to examine the genetic substrates of AUD and its development across the life span.^19^ AUD probands were recruited primarily from treatment facilities across 7 US collection sites. Families of probands that included 3 or more individuals with alcohol dependence were also recruited and included members with and without alcohol dependence. Comparison families (ie, without ascertainment for alcohol dependence or exclusion for it) were also selected from a variety of community sources.^20^ The institutional review boards at all 7 sites approved this study, and written consent was obtained from all participants. We followed the Strengthening the Reporting of Observational Studies in Epidemiology (STROBE) reporting guidelines for cohort studies. Data were restricted to alcohol-exposed individuals (ie, endorsing lifetime use of any alcohol) from 2 COGA subsamples: (1) a cross-sectional cohort of 13 110 individuals from 2234 families assessed from 1991 to 2005, and (2) a longitudinal cohort comprising 2818 offspring of individuals from the cross-sectional cohort, born after 1981 and assessed from 2004 to 2019.^20^

### Measures

#### Cross-Sectional Cohort

The cross-sectional cohort was used to (1) examine correlates of mild, moderate, mild-to-moderate, and severe AUD; (2) identify AUD criteria indicative of heightened risk using IRT analysis; and (3) evaluate whether individuals with mild-to-moderate AUD who endorsed high-risk criteria differed from those who did not, and from those with severe AUD, across relevant factors, including alcohol-related, comorbid psychiatric, and electroencephalography (EEG)-derived traits and polygenic indices. Given extant literature demonstrating substantive differences between severe AUD and mild or moderate AUD,^16,17,18^ and statistical considerations for examining effects of criteria heterogeneity separately within mild and moderate AUD (ie, reduced power, large number of statistical tests^17^), we elected to combine mild and moderate AUD into a single referent group for analyses in both cohorts.

AUD criteria, diagnoses, and several correlates were derived from the Semi-Structured Interview for the Genetics of Alcoholism (SSAGA).^21,22^ Sociodemographic variables included sex, race and ethnicity, current income, educational attainment, and relationship status. Psychiatric lifetime *Diagnostic and Statistical Manual of Mental Disorders* (Fourth Edition) (*DSM-IV*)^23^ diagnoses included major depressive disorder (MDD), antisocial personality disorder (ASPD), and other SUD diagnoses (endorsing ≥2 *DSM-5* criteria for cannabis, cocaine, opiate, stimulant, sedative, or other use disorder or *DSM-IV* nicotine dependence). In addition, several alcohol-related measures were included as correlates in cross-sectional analyses: (1) lifetime endorsement of drinking every day for a week or more, (2) largest number of drinks consumed each day during this period, (3) lifetime endorsement of experiencing blackouts, (4) age at first intoxication, (5) age at regular drinking onset (ie, drinking once per month for 6 months or more), (6) lifetime maximum number of drinks ever consumed in a single 24-hour period, and (7) lifetime endorsement of seeking professional help or engaging in treatment for drinking problems.

COGA includes EEG-derived event-related oscillation (ERO) response measures.^24^ Prior studies have found the P300 component during the standard visual oddball paradigm to be associated with family history of AUD.^24^ Subgroup differences for parietal delta (1-3 Hz) and frontal theta (3-7 Hz) band EROs (300-700 millisecond window) and parietal P300 amplitude were also included as correlates of AUD severity in cross-sectional analyses. Polygenic scores (PGS) for AUD diagnostic status (ie, case/control), derived from a meta-analysis of large-scale genome-wide association study summary statistics,^25,26,27,28^ were calculated for genotyped individuals of European (n = 5396) and African American (n = 1774) ancestry separately using PRS-CS-auto^29^ and PRS-CSx,^30^ respectively (eMethods in Supplement 1).

#### Longitudinal Cohort

The longitudinal cohort included adolescent and young adult participants who were followed up approximately every 2 years (mean [SD] number of time points, 3.2 [1.8]). The SSAGA was administered at each biennial assessment. Given the longitudinal design, this sample was used to examine whether prior mild-to-moderate AUD diagnoses, with and without endorsement of high-risk criteria, were associated with increased hazards of progression to severe AUD. Other well-studied correlates assessed via the SSAGA, including alcohol involvement milestones (ie, age at first drink, regular drinking, and intoxication), and MDD, ASPD, and other SUD diagnoses were also examined.

### Statistical Analyses

#### Cross-Sectional Cohort

**Figure 1.**
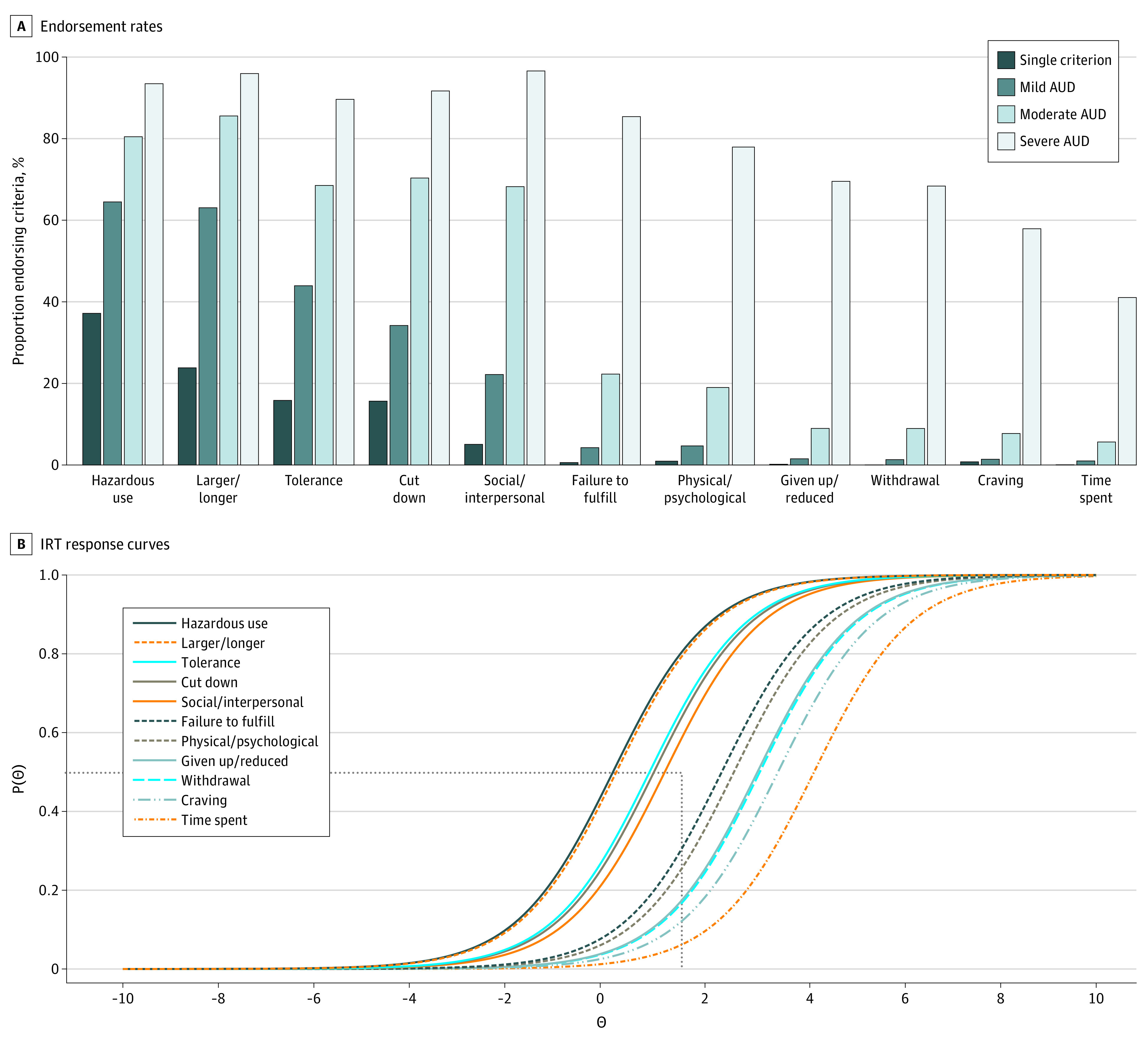
Cross-Sectional Collaborative Study on the Genetics of Alcoholism (COGA) Cohort (N = 13 110) Endorsement Rates and Item Response Theory (IRT) Response Curves for 11 Lifetime Alcohol Use Disorder (AUD) Criteria B, The probability of endorsement of each AUD criteria, P(θ) (y-axis), is plotted as a function of increasing severity of the underlying AUD latent trait, θ (x-axis). The horizontal dotted line represents a 50% probability of endorsing criteria; the vertical dotted line represents 2 SD above the mean of AUD latent severity. Criteria with difficulty parameters of 2 or above (ie, to the right of intersection of vertical and horizontal lines: Failure to fulfill, Physical/psychological, Craving, Withdrawal, Given up/reduced, and Time spent) were identified as high-risk diagnostic criteria. Hazardous use refers to recurrent alcohol use (≥3 times) in situations in which it is physically hazardous; Larger/longer = drinking in larger amounts or over longer periods than intended; Tolerance = need for markedly increased amounts of alcohol to achieve intoxication or desired effect or a markedly diminished effect with continued use of the same amount of alcohol; Cut down = persistent desire or 3 or more unsuccessful efforts to stop, cut down, or control drinking; Social/interpersonal = continued alcohol use despite having persistent or recurrent (≥3 times) social or interpersonal problems caused or exacerbated by the effects of alcohol; Failure to fulfill = recurrent use of alcohol resulting in a failure to fulfill major role obligations at work, school, or home; Physical/psychological = continued drinking despite knowledge of having a persistent or recurrent physical or psychological problem that is likely to be caused or exacerbated by drinking; Craving = craving or a strong desire or urge to use alcohol; Given up/reduced = important social, occupational, or recreational activities given up or reduced because of drinking; Withdrawal = the characteristic withdrawal syndrome for alcohol or drinking (or using a closely related substance) to relieve or avoid withdrawal symptoms; Time spent = a great deal of time spent in activities necessary to obtain, use, or recover from the effects of drinking.

First, descriptive statistics were estimated for sociodemographic, alcohol-related, and comorbid psychiatric measures. Means and proportions were calculated for continuous and categorical variables, respectively, and compared across AUD severity categories. Statistical comparisons of mild vs moderate AUD and moderate vs severe AUD were conducted across correlate measures using Wilcoxon rank sum and Fisher exact tests to evaluate the validity of combining mild and moderate as mild-to-moderate AUD. Second, a 1-parameter logistic IRT model assuming unidimensional structure for the 11 AUD criteria was applied to estimate criteria severity using the mirt package (version 1.37.1)^31^ in R^32^ (eMethods in Supplement 1). Criteria were rank ordered by severity, and those with severity parameter values greater than 2 (ie, 50% endorsement probability by individuals ≥2 SD above mean AUD latent severity) were considered high risk (Figure 1 and eTable 1 in Supplement 1). Third, individuals with mild-to-moderate AUD either endorsing no high-risk criteria or at least 1 high-risk criterion were classified into low-risk (n = 2486) and high-risk (n = 993) groups, respectively. Low-risk mild-to-moderate, high-risk mild-to-moderate, and severe AUD groups were then compared across alcohol-related, psychiatric, and EEG correlates using mixed-effects logistic and linear regression models fitted using the lme4 package (version 1.1-30)^33^ in R controlling for sex, age, self-declared race and ethnicity, and criterion count. Criterion count was included as a covariate given our combined grouping of mild and moderate AUD and our goal to examine associations independent of or not simply due to individual differences in the number of criteria endorsed. Mixed-effects models adjusted for familial clustering. Similar analyses, conducted separately by genetic ancestry, were used to examine associations between AUD PGS and diagnostic groups (eMethods in Supplement 1).

#### Longitudinal Cohort

Hazards of progression to severe AUD (≥6 criteria) were estimated for individuals who, at any previous time point, had endorsed a single criterion or met criteria for mild, moderate, or mild-to-moderate AUD via Cox proportional hazards survival analyses conducted using the survival (version 3.4-0)^34^ and adjustedCurves (version 0.9.1)^35^ packages in R. Low-risk (n = 699) and high-risk (n = 317) mild-to-moderate AUD subgroups were defined by a prior mild-to-moderate AUD diagnosis and differentiated by endorsement of 1 or more high-risk criteria identified in the cross-sectional IRT analysis. Additional survival analyses were conducted examining hazards of progression to severe AUD based on alcohol involvement milestones, MDD, ASPD, and other SUD diagnoses. Covariates for all models included sex, race and ethnicity, and criterion count to examine associations independent of individual differences in the number of prior criteria endorsed. A family grouping variable was used to estimate robust standard errors to account for familial clustering.^36^ Violations of the proportional hazards assumption for predictor variables were tested using Schoenfeld residuals and resolved by including an interaction term with age at onset of severe AUD. Statistical analyses were conducted from December 2022 to June 2023.

## Results

Participants included 13 110 individuals from the cross-sectional COGA cohort (mean [SD] age, 37.8 [14.2] years, 52.8% female) and 2818 individuals from the longitudinal COGA cohort (mean baseline [SD] age, 16.1 [3.2] years; range, 11-26 years; 52.5% female).^20^

### Differences Across Criterion Count–Based AUD Groups

As expected, increasing criterion count (ie, single criterion, mild, moderate, and severe AUD) was associated with increasing levels of heavy alcohol use and greater psychiatric comorbidity (Table 1). For instance, 48.9% and 82.4% of individuals with mild-to-moderate and severe AUD, respectively, reported experiencing blackouts. Likewise, 80% of those with severe AUD met criteria for a comorbid SUD vs half of those with mild-to-moderate AUD. Count-based severity was also reflected in reduced P300 amplitude and theta and delta EROs in individuals with severe vs mild-to-moderate AUD. AUD PGS also differentiated between severe and no AUD in the European ancestry subsample (odds ratio [OR], 1.23; 95% CI, 1.12-1.35) and between severe and mild AUD in the African American ancestry subsample (OR, 1.27; 95% CI, 1.07-1.51) (eTable 3 in Supplement 1). Individuals with mild AUD differed from those with moderate AUD on alcohol-related and psychiatric variables, but overall differences between these 2 severity groups were less pronounced than those between moderate and severe AUD or between mild-to-moderate and severe AUD. The extent of observed differences between mild and moderate AUD, as opposed to severe AUD, and evidence for this pattern in the literature^17^ supported our combined mild-to-moderate group as a comparison with severe AUD.

**Table 1.** Cross-Sectional COGA Cohort (n = 13 110) Descriptive Statistics for Sociodemographic, Alcohol-Related, Psychiatric Comorbidity, Electroencephalography, and AUD Polygenic Score Associations Organized by Diagnostic Group^a^

Variables	% (95% CI)					
	No criteria (n = 4684)	Single criterion (n = 1649)	Mild AUD (2-3 criteria) (n = 2184)	Moderate AUD (4-5 criteria) (n = 1295)	Mild-to-moderate AUD (n = 3479)	Severe AUD (≥6 criteria) (n = 3298)
Sex						
Male	28.0 (26.7-29.3)	43.7 (41.3-46.1)	52.7 (50.6-54.8)	60.3 (57.6-62.9)	55.5 (53.9-57.2)	67.3 (65.6-68.8)
Female	72.0 (70.7-73.3)	56.3 (53.9-58.7)	47.3 (45.2-49.4)	39.7 (37.1-42.4)	44.5 (42.8-46.1)	32.7 (31.2-34.4)
Race and ethnicity						
African American or Black	26.8 (25.6-28.1)	18.2 (16.4-20.1)	17.2 (15.7-18.9)	20.5 (18.4-22.8)	18.5 (17.2-19.8)	24.0 (22.6-25.5)
Asian	1.1 (0.8-1.5)	0.7 (0.4-1.3)	0.5 (0.3-0.9)	0.6 (0.3-1.2)	0.5 (0.3-0.9)	0.6 (0.4-0.9)
Hispanic	7.2 (6.5-8.0)	6.5 (5.4-7.8)	5.7 (4.8-6.7)	5.3 (4.2-6.7)	5.5 (4.8-6.4)	6.1 (5.3-7.0)
Other^b^	2.4 (2.0-2.9)	2.0 (1.4-2.8)	1.6 (1.2-2.3)	2.9 (2.1-3.9)	2.1 (1.7-2.6)	2.6 (2.1-3.2)
Unknown	0.0 (0.0-0.2)	0	0	0	0	0.1 (0.0-0.2)
White	69.6 (68.3-70.9)	79.1 (77.0-81.0)	80.6 (78.9-82.2)	76.0 (73.6-78.2)	78.9 (77.5-80.2)	72.7 (71.2-74.2)
Income (median range), $	30 000-39 999	30 000-39 999	30 000-39 999	30 000-39 999	30 000-39 999	20 000-29 999
Education, mean (SD), y	13.0 (2.3)	13.2 (2.2)	13.1 (2.3)	12.8 (2.2)	13.0 (2.3)	12.2 (2.2)
Relationship						
Married or living as married	52.1 (50.6-53.5)	52.9 (50.5-55.3)	49.1 (47.0-51.2)	43.5 (40.8-46.2)	47.0 (45.3-48.6)	33.3 (31.7-34.9)
Never married	31.2 (29.9-32.6)	33.4 (31.1-35.7)	36.7 (34.6-38.7)	37.4 (34.8-40.1)	36.9 (35.3-38.6)	33.8 (32.2-35.4)
Separated or divorced	12.7 (11.8-13.7)	12.5 (11.0-14.2)	13.0 (11.6-14.5)	17.2 (15.2-19.4)	14.6 (13.4-15.8)	31.4 (29.8-33.0)
Widowed	4.0 (3.5-4.6)	1.2 (0.8-1.9)	1.3 (0.9-1.9)	1.9 (1.3-2.9)	1.5 (1.2-2.0)	1.6 (1.2-2.1)
Alcohol-related						
Drinking every day for ≥1 wk	16.8 (15.4-18.2)	32.4 (30.2-34.7)	53.1 (51.0-55.2)	74.1 (71.6-76.4)	60.9 (59.3-62.5)	93.6 (92.7-94.4)
No. of drinks every day for ≥1 wk, mean (SD)^c^	3.1 (3.0)	4.4 (4.0)	6.4 (6.1)	8.8 (8.9)	7.5 (7.6)	16.1 (13.5)
Experienced blackouts	11.2 (10.1-12.5)	25.0 (23-27.2)	41.3 (39.3-43.4)	61.7 (59.0-64.3)	48.9 (47.3-50.6)	82.4 (81.1-83.7)
Age at first intoxication, mean (SD), y	20.1 (6.6)	17.8 (4.6)	17.0 (4.3)	16.4 (4.1)	16.7 (4.3)	15.3 (4.4)
Age at regular drinking, mean (SD), y	22.1 (7.7)	19.5 (5.0)	18.7 (5.0)	18.2 (5.0)	18.5 (5.0)	17.1 (4.9)
Maximum No. of drinks, mean (SD)^d^	6.6 (6.6)	12.5 (9.5)	16.6 (11.5)	22.0 (15.0)	18.6 (13.2)	34.1 (20.0)
Sought help/treatment	0.7 (0.4-1.0)	2.8 (2.1-3.7)	8.8 (7.7-10.1)	28.1 (25.7-30.6)	16.0 (14.8-17.2)	79.4 (78.0-80.8)
Psychiatric comorbidity						
MDD	14.0 (12.8-15.3)	14.0 (12.0-16.2)	15.4 (13.7-17.4)	13.2 (11.1-15.8)	14.7 (13.2-16.2)	23.3 (21.4-25.4)
ASPD^e^	2.5 (2.1-3.0)	5.5 (4.4-6.7)	8.1 (7.0-9.3)	13.5 (11.7-15.5)	10.1 (9.1-11.1)	24.4 (22.9-25.9)
SUD^f^	16.7 (15.6-17.7)	31.8 (29.6-34.1)	43.5 (41.4-45.6)	59.2 (56.5-61.9)	49.4 (47.7-51.0)	80.0 (78.6-81.4)
Theta ERO, mean (SD)	26.7 (18.1)	25.9 (17.1)	27.1 (17.6)	23.8 (14.1)	25.8 (16.5)	20.5 (14.0)
Delta ERO, mean (SD)	49.3 (29.5)	49.4 (28.8)	49.6 (36.5)	42.8 (21.3)	47 (31.8)	38.1 (22.4)
P300 amplitude, mean (SD)	17.5 (9.4)	18 (8.9)	18.2 (9.5)	16.8 (8.6)	17.6 (9.2)	13.9 (8.3)
AUD PGS, % in top quintile (95% CI)^g^	16.8 (15.3-18.3)	17.5 (15.1-20.2)	20.4 (18.2-22.7)	23.4 (20.5-26.5)	21.5 (19.8-23.4)	23.4 (21.6-25.3)

Abbreviations: ASPD, antisocial personality disorder; AUD, alcohol use disorder; COGA, Collaborative Study on the Genetics of Alcoholism; *DSM-IV* and *DSM-5*, *Diagnostic and Statistical Manual of Mental Disorders*, Fourth Edition and Fifth Edition; ERO, event-related oscillation; MDD, major depressive disorder; PGS, polygenic score; SUD, comorbid substance use disorder.

^a^ Comparison sample sizes varied across correlates according to patterns of missing data (eTable 2 in Supplement 1).

^b^ Other race and ethnicity comprises Native American, Pacific Islander, and self-declared “other” race.

^c^ Sample sizes for maximum number of drinks consumed every day during period of drinking every day for ≥1 week are restricted based on endorsement of ever drinking every day for ≥1 week.

^d^ Maximized over available interviews.

^e^ Nonsignificant difference between odds ratio for mild vs moderate AUD and odds ratio for moderate vs severe AUD. For all alcohol-related and psychiatric comorbidity variables, rank-biserial correlations from Wilcoxon rank sum tests for continuous measures were converted to odds ratios and compared with odds ratios calculated from Fisher exact tests for dichotomous measures. Significant differences in odds ratios were calculated using *z* statistics computed from log odds differences divided by pooled standard error estimates. All other comparisons were significant at 2-tailed *P* < .05 with differences between moderate and severe AUD being larger than differences between mild and moderate AUD.

^f^ *DSM-5* cannabis use disorder, cocaine use disorder, opiate use disorder, simulant use disorder, sedative use disorder, and other drug use disorder and *DSM-IV* nicotine dependence.

^g^ Based on AUD PGS quintiles calculated separately by ancestral subsample prior to combined presentation.

### High- and Low-Risk Criteria

IRT analysis of the 11 *DSM-5* criteria in the cross-sectional cohort revealed that failure to fulfill major role obligations because of drinking, drinking despite physical/psychological problems, giving up/reducing important activities, withdrawal, craving, and spending a great deal of time drinking represented more severe criteria, indexing greater risk (Figure 1 and eTable 1 in Supplement 1). Endorsement of these 6 high-severity criteria differed considerably across criterion count–based severity groups (single criterion, mild, moderate, and severe AUD). For instance, withdrawal was endorsed by 4.2% of individuals with mild-to-moderate AUD and, in contrast, by 68.3% of those with severe AUD. As expected, individuals with mild AUD were less likely to endorse high-risk criteria (eg, 1.3% endorsing withdrawal) compared with those with moderate AUD (eg, 9.0% endorsing withdrawal).

### High- and Low-Risk Mild-to-Moderate AUD vs Severe AUD

Individuals with high-risk mild-to-moderate AUD were more likely to endorse a greater number of criteria than those with low-risk mild-to-moderate AUD (eg, 42.5% vs 6.8% endorsed 5 criteria). Even after accounting for the number of criteria endorsed, individuals with mild-to-moderate AUD who endorsed high-risk criteria were more likely to consume a greater number of drinks during heavy drinking episodes and periods of frequent drinking; endorse seeking help or treatment; meet criteria for other SUDs, MDD, and ASPD; and have lower theta EROs when compared with individuals with low-risk mild-to-moderate AUD (Table 2 and eTable 5 in Supplement 1). AUD PGS also distinguished between low-risk mild-to-moderate and severe AUD in the African American ancestry subsample (OR, 1.30; 95% CI, 1.09-1.56) (eTable 3 in Supplement 1). Notably, the high-risk mild-to-moderate group did not statistically differ from the severe AUD group on theta EROs or P300 amplitude after accounting for criterion count differences.

**Table 2.** Cross-Sectional COGA Cohort Comparisons of Alcohol-Related, Psychiatric Comorbidity, Electroencephalography, and AUD Polygenic Score Associations by Low- and High-Risk Mild-to-Moderate and Severe AUD^a^

Variables	Mean (SD)		
	Mild-to-moderate AUD		Severe AUD (n = 3298)
	Endorsed only low-risk criteria (n = 2486)	Endorsed ≥1 high-risk criteria (n = 993)	
Alcohol-related			
Drinking every day for ≥1 wk, % (95% CI)^b^	57.2 (55.2-59.1)	70.2 (67.3-73.0)	93.6 (92.7-94.4)
No. of drinks every day for ≥1 wk^b^^,^^c^^,^^d^	6.6 (5.9)	9.4 (10.1)	16.1 (13.5)
Experienced blackouts, % (95% CI)^b^	46.1 (44.1-48.1)	56 (52.9-59.1)	82.4 (81.1-83.7)
Age at first intoxication, y^b^	16.8 (3.9)	16.7 (5.1)	15.3 (4.4)
Age at regular drinking, y^b^	18.5 (4.7)	18.5 (5.8)	17.1 (4.9)
Maximum No. of drinks^b^^,^^c^^,^^e^	17.5 (11.4)	21.5 (16.4)	34.1 (20.0)
Sought help/treatment, % (95% CI)^b^^,^^c^	11.3 (10.2-12.7)	27.6 (24.9-30.5)	79.4 (78.0-80.8)
Psychiatric comorbidity, % (95% CI)			
MDD^b^^,^^c^	13.9 (12.3-15.7)	16.7 (13.9-19.9)	23.3 (21.4-25.4)
ASPD^b^^,^^c^	8.2 (7.1-9.3)	15.0 (12.9-17.5)	24.4 (22.9-25.9)
SUD^b^^,^^c^^,^^f^	44.4 (42.5-46.4)	61.7 (58.7-64.7)	80.0 (78.6-81.4)
Theta ERO^c^	27.0 (17.3)	22.9 (13.9)	20.5 (14.0)
Delta ERO	48.3 (33.8)	43.7 (25.9)	38.1 (22.4)
P300 amplitude	18.3 (9.3)	15.9 (8.6)	13.9 (8.3)
AUD PGS, % in top quintile (95% CI)	21.0 (19.0-23.2)	22.8 (19.5-26.4)	23.4 (21.6-25.3)
African American ancestry subsample	18.6 (13.8-24.6)	19.5 (14.4-25.8)	22.3 (18.9-26.1)
European ancestry subsample	21.4 (19.2-23.8)	24.2 (20.4-29.0)	23.8 (21.7-26.1)

Abbreviations: ASPD, antisocial personality disorder; AUD, alcohol use disorder; COGA, Collaborative Study on the Genetics of Alcoholism; *DSM-IV* and *DSM-5*, *Diagnostic and Statistical Manual of Mental Disorders*, Fourth Edition and Fifth Edition; ERO, event-related oscillation; MDD, major depressive disorder; PGS, polygenic score; SUD, comorbid substance use disorder.

^a^ Comparison sample sizes varied across correlates according to patterns of missing data (eTable 4 in Supplement 1).

^b^ Denotes variables exhibiting significant differences (*P* < .05) between high-risk mild-to-moderate and severe AUD in mixed models comparing low-risk and high-risk mild-to-moderate AUD with severe AUD (reference group) controlling for age, sex, race and ethnicity, and criterion count and nested within family (for alcohol-related, psychiatric, and electroencephalography correlates) or age, age^2^, sex, 10 ancestral principal components, genotyping array (for European ancestry subsample), cohort, and nested within family (for PGS correlates).

^c^ Denotes variables exhibiting significant differences (*P* < .05) between low-risk and high-risk mild-to-moderate AUD in mixed models comparing low-risk mild-to-moderate and severe AUD with high-risk mild-to-moderate AUD (reference group) controlling for age, sex, race and ethnicity, and criterion count and nested within family (for alcohol-related, psychiatric, and electroencephalography correlates) or age, age^2^, sex, 10 ancestral principal components, genotyping array (for European ancestry subsample), cohort, and nested within family (for PGS correlates).

^d^ Sample sizes for maximum number of drinks consumed every day during period of drinking every day for ≥1 week are restricted based on endorsement of ever drinking every day for ≥1 week.

^e^ Maximized over available interviews.

^f^ *DSM-5* cannabis use disorder, cocaine use disorder, opiate use disorder, simulant use disorder, sedative use disorder, and other drug use disorder and *DSM-IV* nicotine dependence.

### Hazards of Progression to Severe AUD

**Figure 2.**
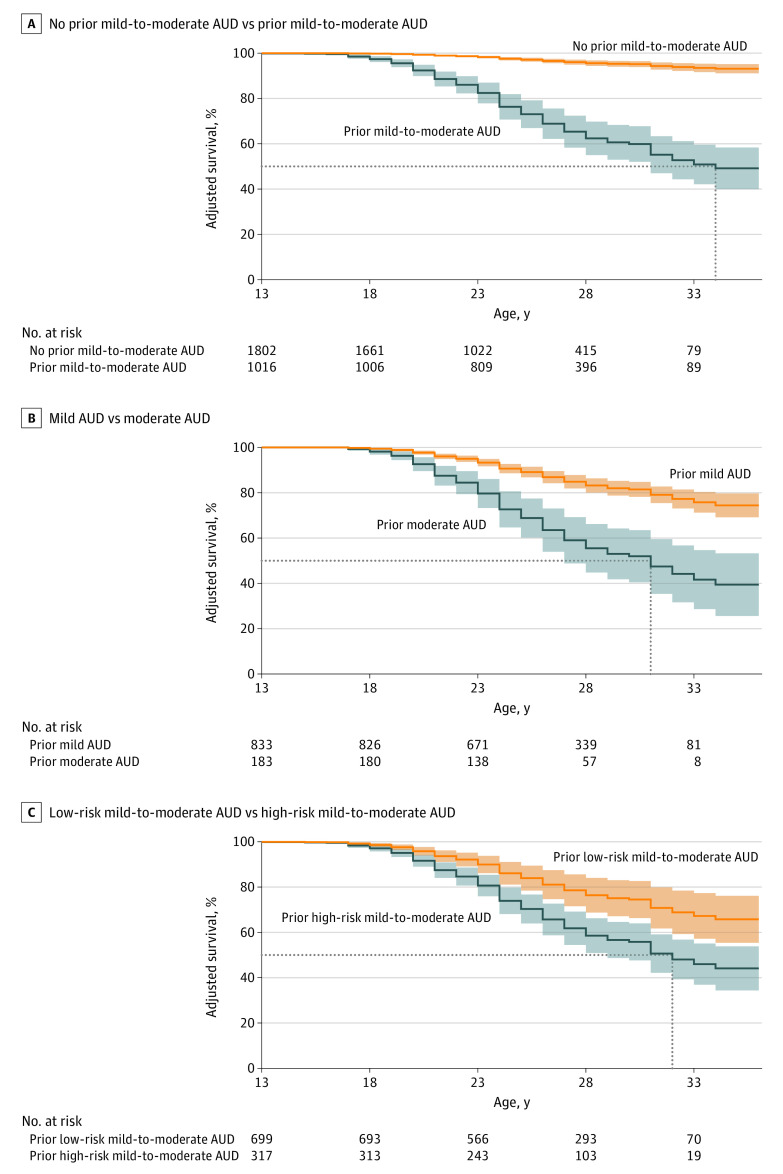
Longitudinal Collaborative Study on the Genetics of Alcoholism (COGA) Cohort (N = 2818) Survival Curves and 95% Confidence Intervals for Progression to Severe Alcohol Use Disorder (AUD) All survival curves include no prior mild-to-moderate AUD as comparison and are adjusted for sex, race and ethnicity, and mild-to-moderate AUD criterion count. A, Survival curves additionally adjusted for endorsement of prior mild-to-moderate AUD. B, Survival curves additionally adjusted for mild vs moderate AUD. C, Survival curves additionally adjusted for low-risk vs high-risk mild-to-moderate AUD. Dotted lines represent point estimates of median survival ages (not accounting for 95% CIs): A, prior mild-to-moderate AUD = 34 years; B, mild AUD = undefined, moderate AUD = 31 years; C, low-risk mild-to-moderate AUD = undefined, high-risk mild-to-moderate AUD = 32 years.

A majority of individuals who met criteria for severe AUD (77.8%) had a history of mild-to-moderate AUD (9.5% endorsed a single prior criterion, 12.7% endorsed no prior criteria). Consistent with cross-sectional findings, individuals with high-risk mild-to-moderate AUD were more likely (33.4%) to transition to severe AUD than those with low-risk mild-to-moderate AUD (12.9% transitioned to severe AUD) (Table 3 and eResults in Supplement 1). The hazard of transitioning from high-risk mild-to-moderate AUD to severe AUD (adjusted hazard ratio [aHR], 11.62; 95% CI, 7.54-17.92) was more than double that of transitioning to severe AUD from low-risk mild-to moderate AUD after accounting for criterion count (aHR, 5.64; 95% CI, 3.28-9.70; between-group aHR, 2.06; 95% CI, 1.47-2.88) (Figure 2). Earlier ages at first drink, regular drinking, and first intoxication and comorbid ASPD, MDD, and SUDs were significantly associated with progression to severe AUD; however, hazards for these characteristics were considerably lower than hazards for belonging to the high-risk mild-to-moderate AUD group. In multivariate models, high-risk mild-to-moderate was the strongest predictor of progression to severe AUD (aHR, 4.25; 95% CI, 2.57-7.04).

**Table 3.** Longitudinal COGA Cohort (n = 2818) Results From Cox Proportional Hazards Models for Progression to Severe AUD^a^

	No criteria	Prior single criterion	Prior mild AUD (2-3 criteria)	Prior moderate AUD (4-5 criteria)	Prior mild-to-moderate		
					Endorsed only low-risk criteria	Endorsed ≥1 high-risk criterion	Total
Sample size, No.	1236	566	833	183	699	317	1016
Proportion progressing to severe AUD (≥6 criteria), % (95% CI)	2.6 (1.8-3.6)	4.2 (2.9-6.3)	15.4 (13.1-18.0)	37.2 (30.4-44.4)	12.9 (10.6-15.6)	33.4 (28.5-38.8)	19.3 (17.0-21.8)
Adjusted Cox proportional hazards ratio (95% CI)	NA	1.13 (0.66-1.93)	3.43 (2.33-5.05)	11.10 (7.17-17.20)	5.64 (3.28-9.70)	11.62 (7.53-17.92)	11.30 (7.33-17.43)

Abbreviations: AUD, alcohol use disorder; COGA, Collaborative Study on the Genetics of Alcoholism; NA, not applicable.

^a^ All Cox proportional hazards models included sex and race and ethnicity as covariates clustered within family. Mild and moderate AUD models additionally included dummy-coded variables for criterion count (ie, 0/1 for 2 vs 3 criteria for mild; 0/1 for 4 vs 5 criteria for moderate). Mild-to-moderate AUD models additionally included dummy-coded variables for criterion count for 2 to 5 criteria.

## Discussion

We sought to examine whether endorsement of certain diagnostic criteria was associated with higher risk for severe AUD within the *DSM-5* diagnostic scheme. IRT analyses revealed 6 high-risk criteria that reflect greater severity, and even after accounting for increasing criterion counts, there were significant differences among individuals who endorsed high- vs low-risk criteria. Moreover, individuals with mild-to-moderate AUD who endorsed 1 of these high-risk criteria were statistically indistinguishable from those with severe AUD with respect to theta EROs and P300 amplitude (after accounting for differences in criterion count). Individuals with moderate or high-risk mild-to-moderate AUD had the greatest hazards of progression to severe AUD across late adolescence and early adulthood. Overall, these analyses suggest that the presence of specific criteria are a superior indicator of risk for progression to severe AUD compared with criterion count alone. This difference is especially pronounced for the mild-to-moderate AUD group.

Many of the 6 empirically identified high-risk criteria have previously demonstrated stronger associations with comorbid psychopathology and greater severity of and discriminatory capabilities for AUD than other criteria.^13,37,38,39^ However, these high-risk criteria do not currently comprise a coherent *DSM-5* AUD subtype.^40^ For example, although *DSM-5* characterizes both withdrawal and tolerance as physiological components of AUD, withdrawal but not tolerance was identified as high risk. Interestingly, several of the high-risk criteria were markers of preoccupation (eg, craving, time spent, giving up/reducing important activities) and impairment in several domains (eg, role obligations, recurrent physical/psychological problems, withdrawal). These criteria were endorsed far more frequently by individuals with severe AUD and map onto 2 key stages (preoccupation/anticipation and withdrawal/negative affect) of one of the neurobiological models developed to characterize severe forms of SUDs.^41^

Unlike heavy drinking or psychiatric comorbidity, which may arise as a consequence of problematic drinking patterns, PGS and EEG measures provide a glimpse into potential and preexisting neurobiological vulnerabilities. EEG and AUD PGS results generally echoed other findings. Specifically, theta EROs distinguished between individuals with low-risk and high-risk mild-to-moderate AUD and demonstrated similarly blunted neurophysiological responses in both high-risk mild-to-moderate and severe AUD groups in the cross-sectional cohort. AUD PGS primarily exhibited associations with criterion count and overall diagnostic status (AUD vs no AUD) across both ancestry subsamples. As these PGS are calculated using genome-wide association studies of broadly conceptualized AUD status often obtained from electronic health record–derived diagnostic codes,^25,26,27,28^ findings here highlight the need for criterion-focused genome-wide association studies to resolve key genetic mechanisms that might underlie specific clinical presentations.^38,39,42,43,44^

In a recent conceptual proposal, McLellan et al^45^ note that while individuals with mild-to-moderate SUDs are currently not a high-priority population for treatment efforts, they represent “one reasonable starting point” for defining a state similar to prediabetes, indexing accumulating risk for progression to severe SUD diagnosis, and thus merit heightened vigilance and intervention. While the proposed concept has drawn criticism regarding potentially stigmatizing terminology,^46,47,48^ the importance of improving treatment efforts for all presentations of AUD cannot be overstated.^6^ In the current study, despite heightened risk, only 27.6% of the high-risk mild-to-moderate AUD group endorsed seeking professional help or engaging in treatment, which, although significantly more than rates endorsed by individuals in the low-risk mild-to-moderate AUD group, is far from ideal.

Our study provides mixed support for combining across mild and moderate AUD. In the cross-sectional cohort, differences between severe and moderate AUD were more pronounced than between moderate and mild AUD. However, endorsement of high-risk criteria was notably lower in those with mild vs moderate AUD, and in the longitudinal cohort, hazards of progressing to severe AUD from mild or low-risk mild-to-moderate AUD were much lower than hazards for moderate or high-risk mild-to-moderate AUD, which were largely equivalent. These findings are generally consistent with research suggesting that mild AUD may more accurately reflect temporally limited drinking problems consistent with endorsement of less severe criteria.^49,50,51,52^

### Limitations

The current study should be interpreted in light of its limitations. First, identification of high-risk criteria may be influenced by the AUD-enriched family-based study design, though comparisons with prior IRT estimates suggest consistent results.^13,14^ Notwithstanding, findings here may not generalize to samples more representative of the general population. Second, although the cross-sectional cohort has a wide age distribution, longitudinal data are delimited to younger ages. Third, many *DSM-5* criteria assessed using the SSAGA are compound criteria comprising multiple items of varying severity (eg, social/interpersonal problems: objections from others vs loss of friends). Disaggregation of compound criteria into individual items, though not always practical in clinical settings, may help further elucidate symptoms signaling increased risk for development of severe AUD.^53^

## Conclusions

In this cohort study involving a combined 15 928 individuals, findings suggested that certain high-risk criteria may improve the identification of individuals at higher risk of progression to severe AUD. Defining severe AUD vulnerability using these criteria is consistent with extant etiological and clinical insight and may improve treatment penetrance.

## Notes

Supplement 1. **eMethods.** Supplemental methods **eTable 1.** Cross-sectional COGA cohort (*N* = 13,110) item response theory severity parameters and endorsement rates for DSM-5 AUD criteria organized by diagnostic groups **eTable 2.** All correlate sample sizes for COGA cross-sectional cohort organized by diagnostic groups **eTable 3.** Results from mixed effect logistic AUD PGS regression models in COGA cross-sectional sample **eTable 4.** Alcohol-related, psychiatric comorbidity, electroencephalography, and AUD polygenic score correlate sample sizes for COGA cross-sectional cohort organized by low- and high-risk mild-to-moderate and severe AUD **eTable 5.** Results from mixed linear and logistic regression models comparing alcohol-related, psychiatric, electroencephalography, and polygenic score correlates across low-risk mild-to-moderate, high-risk mild-to-moderate, and severe AUD in COGA cross-sectional cohort **eResults.** Supplemental survival analysis results
eReferences

Supplement 2.
Nonauthor Collaborators

Supplement 3.
Data Sharing Statement
