# Supplementary material for: Diagnostic Criteria for Identifying Individuals at High Risk of Progression From Mild or Moderate to Severe Alcohol Use Disorder
Source: JAMA Netw Open. 2023 Oct 10;6(10):e2337192. doi: 10.1001/jamanetworkopen.2023.37192 (PMC10565602; doi:10.1001/jamanetworkopen.2023.37192)
Supplement: Supplement 1. — eMethods. Supplemental methods eTable 1. Cross-sectional COGA cohort (N = 13,110) item response theory severity parameters and endorsement rates for DSM-5 AUD criteria organized by diagnostic groups eTable 2. All correlate sample sizes for COGA cross-sectional cohort organized by diagnostic groups eTable 3. Results from mixed effect logistic AUD PGS regression models in COGA cross-sectional sample eTable 4. Alcohol-related, psychiatric comorbidity, electroencephalography, and AUD polygenic score correlate sample sizes for COGA cross-sectional cohort organized by low- and high-risk mild-to-moderate and severe AUD eTable 5. Results from mixed linear and logistic regression models comparing alcohol-related, psychiatric, electroencephalography, and polygenic score correlates across low-risk mild-to-moderate, high-risk mild-to-moderate, and severe AUD in COGA cross-sectional cohort eResults. Supplemental survival analysis results eReferences [file jamanetwopen-e2337192-s001.pdf]

Supplemental Online Content

Miller AP, Kuo SIC, Johnson EC, et al; Collaborative Study on the Genetics of Alcoholism (COGA). Diagnostic criteria for identifying individuals at high risk of progression from mild or moderate to severe alcohol use disorder. *JAMA Netw Open*. 2023;6(10):e2337192. doi:10.1001/jamanetworkopen.2023.37192

eMethods. Supplemental methods
eTable 1. Cross-sectional COGA cohort (N = 13,110) item response theory severity parameters and endorsement rates for DSM-5 AUD criteria organized by diagnostic groups
eTable 2. All correlate sample sizes for COGA cross-sectional cohort organized by diagnostic groups
eTable 3. Results from mixed effect logistic AUD PGS regression models in COGA cross-sectional sample
eTable 4. Alcohol-related, psychiatric comorbidity, electroencephalography, and AUD polygenic score correlate sample sizes for COGA cross-sectional cohort organized by low- and high-risk mild-to-moderate and severe AUD
eTable 5. Results from mixed linear and logistic regression models comparing alcohol-related, psychiatric, electroencephalography, and polygenic score correlates across low-risk mild-to-moderate, high-risk mild-to-moderate, and severe AUD in COGA cross-sectional cohort
eResults. Supplemental survival analysis results
eReferences

This supplementary material has been provided by the authors to give readers additional information about their work.

© 2023 Miller AP et al. *JAMA Network Open.*

eMethods. Supplemental methods

Assessment

Diagnostic assessment of an array of lifetime alcohol use disorder (AUD) symptoms in COGA, which can be mapped onto a variety of diagnostic classification systems, including both DSM-IV[1] and DSM-5[2], was conducted using the Semi-Structured Interview for the Genetics of Alcoholism (SSAGA), which also includes criterion-level and diagnostic assessments of other psychiatric and substance use disorders (SUDs) as well as other non-diagnostic aspects of alcohol use.[3,4] Individuals below age 18 at time of assessment were administered an adolescent SSAGA (c-SSAGA)[4,5] in both cross-sectional and longitudinal cohorts. SSAGA interviews were completed in-person or by telephone and administered by trained interviewers.

Alcohol Use Disorder Polygenic Scores

AUD polygenic scores (PGS) were calculated for genotyped individuals of European (EA; n=5,396) and African-American (AA; n=1,774) ancestry. Ancestral background was defined using both principal components analysis of array data and self-declared non-Hispanic white (EA) and non-Hispanic Black (AA) ethnicity/race. Additional details regarding genotyping, imputation, and quality control are available in a related publication.[6] Summary statistics from a genome-wide association study (GWAS) meta-analysis of data from the Million Veterans Program (MVP; n_{case}=45,995, $n_{control}$=221,396),[7] a "leave-one-out" GWAS of alcohol dependence from the Psychiatric Genomics Consortium (PGC) analysis that excluded the COGA sample (n_{case}=10,381, $n_{control}$=31,801),[8] and FinnGen Research Project Release 8 (n_{case}=14,307, $n_{control}$=328,192)[9] were used to code PGS using PRS-CS-auto[10] in EA individuals. PRS-CSx,[11] was used with GWAS summary statistics from both the EA analyses as well as a GWAS meta-analysis of AUD in AA individuals from PGC, not including COGA (n_{case}=3,335, $n_{control}$=2,945)[8] and MVP (n_{case}=17,267, $n_{control}$=39,381)[12] to create meta-analyzed combined weights. Individual PGSs were then calculated in PLINK 1.9[13] using PRS-CS- and PRS-CSx-derived weights. PGS were scaled to M=0, SD=1 using the *scale* function in R.

Diagnostic group (i.e., low-risk mild-to-moderate, high-risk mild-to-moderate, and severe AUD) associations with AUD PGS were examined separately for each ancestral group using mixed effect linear regression models fitted using the *lme4* package (v1.1-30)[14] controlling for age, age^2, sex, 10 genetic ancestry principal components, birth cohort (dummy variables representing birth years prior to 1930, 1930 to 1949, 1950 to 1969, and 1970 and after), and genotyping array type (for European subsample) as fixed effects, and for family ID as a random intercept. Mixed models were specified comparing (1) low-risk and high-risk mild-to-moderate AUD to severe AUD (reference group) and (2) low-risk mild-to-moderate and severe AUD to high-risk mild-to-moderate AUD (reference group). Additional mixed effect linear regression models with these covariates were used to examine associations between AUD PGSs and AUD criterion counts. Follow-up pairwise comparisons (e.g., high-risk *vs.* low-risk mild-to-moderate AUD, moderate *vs.* mild AUD, AUD *vs.* no AUD, etc.) were also conducted using mixed effect logistic regression models with these same covariates (**eTable 3**).

Item Response Theory Analysis

In the current context, item response theory (IRT) models allow for the investigation of the latent continuum of a disorder (i.e., AUD[15,16]) by establishing a link between the properties of criteria on a diagnostic instrument (e.g., SSAGA criteria 'severity' parameters – point on AUD continuum at which probability of endorsement reaches 0.50), individuals responding to these items (i.e., position along the underlying latent AUD continuum), and the underlying construct (i.e., AUD). Primary assumptions of IRT include (a) local dependence (i.e., responses to each criterion are independent of each other) and (b) unidimensionality (i.e., these criteria approximate a unidimensional construct). When the unidimensionality assumption is supported, the assumption of local independence is also likely to be met.[17] To test the assumptions of local independence and unidimensionality of a one-parameter logistic IRT model in the current study,[17] a criteria-based confirmatory factor analysis was conducted for the full sample specifying a single AUD factor using weighted least square mean and variance adjusted estimation in the *lavaan* package (v0.6-12)[18] in R.[19] This single-factor model provided excellent fit to data (RMSEA, 0.035; 90% CI, 0.033-0.037; CFI, 0.998; TLI, 0.998; SRMR, 0.020). While two-parameter IRT models also provide information regarding the relative discrimination ability of each criteria/item, as has been suggested previously,[15,20] these models significantly complicate the goal of selecting criteria based on severity as they introduce further complexity given

© 2023 Miller AP et al. *JAMA Network Open.*

the additional information regarding discrimination. Thus, for the current approach, the one-parameter logistic model is preferred as it provides a more parsimonious description of the data, provides adequate fit, and is more consistent with current AUD diagnostic schemes in clinical practice (i.e., presence *vs.* absence). In this context, criteria with lower severity parameters are more likely to be endorsed and thus represent less severe criteria. In contrast, criteria with higher severity parameters are less likely to be endorsed and represent more severe criteria.

Survival Analyses

The longitudinal cohort was restricted to alcohol-exposed individuals having data for at least baseline and one follow-up interview. The small number (*n*=71) of individuals who had met criteria for severe AUD at baseline were also excluded. As with the cross-sectional sample, sex, self-reported race/ethnicity, and AUD diagnostic information were measured using SSAGA/c-SSAGA interviews at each available timepoint for each individual in the longitudinal cohort (*N*=2,818). Waves of data were restructured to reflect age at each assessment (range=13-36 years). Age at onset of severe AUD was coded as the earliest assessment age at which an individual endorsed 6+ AUD criteria, while age at final assessment was used for those who did not meet severe AUD criteria during the study period (i.e., right-censored).

Missing Data

For all analyses, missing data were handled using listwise deletion – analytic sample sizes were based on individuals having data for all variables of interest. Sample sizes for each correlate variable and diagnostic group are presented in eTables 2 and 4. For most analyses, missing correlate data was minimal, and in the full cross-sectional cohort, analytic sample sizes ranged from 1,350 individuals for low-risk mild-to-moderate AUD *vs*. high-risk mild-to-moderate AUD on P300 amplitude to 5,784 individuals for low-risk mild-to-moderate AUD *vs*. severe AUD. In the European and African-American ancestry subsamples, PGS model analytic sample sizes ranged from 1,589 for low-risk mild-to-moderate AUD *vs*. high-risk mild-to-moderate AUD to 2,652 for low-risk mild-to-moderate AUD *vs*. severe AUD (European ancestry) and from 389 for low-risk mild-to-moderate AUD *vs*. high-risk mild-to-moderate AUD to 723 for low-risk mild-to-moderate AUD *vs*. severe AUD (African-American ancestry).

eTable 1. Cross-sectional COGA cohort (*N* = 13,110) item response theory severity parameters and endorsement rates for DSM-5 AUD criteria organized by diagnostic groups

	Severity Parameter *b* (SE)	Average Criterion Count *M* (*SD*)	Criteria Endorsement by Diagnostic Group (%, 95% CI)				
				Mild-to-Moderate AUD			
			Single Criterion	Mild AUD (2-3 criteria)	Moderate AUD (4-5 criteria)	Mild-to-Moderate AUD (2-5 criteria)	Severe AUD (≥6 criteria)
			n = 1,649	*n* = 2,184	*n* = 1,295	*n* = 3,479	*n* = 3,298
Hazardous Use	0.07 (0.03)	5.8 (3.3)	37.2 (34.9-39.6)	64.5 (62.4-66.5)	80.5 (78.2-82.5)	70.4 (68.9-71.9)	93.5 (92.6-94.3)
Larger/Longer	0.14 (0.03)	6.0 (3.2)	23.9 (21.9-26.0)	63.0 (61.0-65.1)	85.5 (83.5-87.4)	71.4 (69.9-72.9)	96.0 (95.3-96.6)
Tolerance	0.82 (0.04)	6.5 (3.2)	15.8 (14.1-17.6)	43.9 (41.8-46.0)	68.5 (65.9-71.0)	53.1 (51.4-54.7)	89.7 (88.6-90.7)
Cut Down	0.91 (0.04)	6.7 (3.1)	15.6 (14.0-17.5)	34.2 (32.2-36.2)	70.3 (67.8-72.8)	47.6 (46.0-49.3)	91.7 (90.7-92.6)
Social Interpersonal	1.14 (0.04)	7.2 (2.8)	5.0 (4.1-6.2)	22.2 (20.5-24.0)	68.2 (65.6-70.7)	39.3 (37.7-41.0)	96.6 (95.9-97.2)
**Failure to Fulfill**	**2.31 (0.04)**	**8.4 (2.2)**	**0.6 (0.3-1.1)**	**4.2 (3.4-5.1)**	**22.3 (20.1-24.7)**	**11.0 (10.0-12.0)**	**85.4 (84.1-86.6)**
**Physical/Psychological**	**2.56 (0.04)**	**8.5 (2.3)**	**0.9 (0.5-1.5)**	**4.7 (3.9-5.7)**	**19.0 (17.0-21.2)**	**10.0 (9.1-11.1)**	**77.9 (76.5-79.3)**
**Given up/Reduced**	**3.04 (0.04)**	**9.0 (1.9)**	**0.2 (0.1-0.6)**	**1.6 (1.1-2.2)**	**9.0 (7.5-10.6)**	**4.3 (3.7-5.0)**	**69.5 (67.9-71.0)**
**Withdrawal**	**3.09 (0.04)**	**9.0 (1.9)**	–	**1.3 (0.9-1.9)**	**9.0 (7.5-10.6)**	**4.2 (3.6-4.9)**	**68.3 (66.7-69.9)**
**Craving**	**3.46 (0.04)**	**9.1 (2.0)**	**0.8 (0.5-1.4)**	**1.4 (1.0-2.0)**	**7.7 (6.4-9.3)**	**3.8 (3.2-4.5)**	**57.9 (56.2-59.6)**
**Time Spent**	**4.21 (0.04)**	**9.4 (2.0)**	**0.1 (0.0-0.4)**	**1.0 (0.7-1.5)**	**5.6 (4.5-7.0)**	**2.7 (2.2-3.3)**	**41.0 (39.4-42.7)**

Note: AUD = alcohol use disorder; **Bold** text denotes criteria designated as high-risk based on IRT results with severity parameter values >2 (i.e., 50% endorsement probability by individuals 2+ *SD* above mean AUD latent severity). Hazardous Use = Recurrent alcohol use (3+ times) in situations in which it is physically hazardous; Larger/longer = Drinking in larger amounts or over longer periods than intended; Tolerance = Need for markedly increased amounts of alcohol to achieve intoxication or desired effect or a markedly diminished effect with continued use of the same amount of alcohol; Cut Down = Persistent desire or three or more unsuccessful efforts to stop, cut down, or control drinking; Social Interpersonal = Continued alcohol use despite having persistent or recurrent (3+ times) social or interpersonal problems caused or exacerbated by the effects of alcohol; Failure to Fulfill = Recurrent use of alcohol resulting in a failure to fulfill major role obligations at work, school, or home; Physical/Psychological = Continued drinking despite knowledge of having a persistent or recurrent physical or psychological problem that is likely to be caused or exacerbated by drinking; Craving = Craving, or a strong desire or urge to use alcohol; Given up/Reduced = Important social, occupational, or recreational activities given up or reduced because of drinking; Withdrawal = The characteristic withdrawal syndrome for alcohol; or drinking (or using a closely related substance) to relieve or avoid withdrawal symptoms; Time Spent = A great deal of time spent in activities necessary to obtain, to use, or to recover from the effects of drinking.

© 2023 Miller AP et al. *JAMA Network Open.*

eTable 2. All correlate sample sizes for COGA cross-sectional cohort organized by diagnostic groups

	No Criteria	Single Criterion	Mild AUD (2-3 criteria)	Moderate AUD (4-5 criteria)	Mild-to-Moderate AUD	Severe AUD (≥6 criteria)
Variables	*n* = 4,684	*n* = 1,649	*n* = 2,184	*n* = 1,295	*n* = 3,479	*n* = 3,298
Sociodemographic						
Sex	4,684	1,649	2,184	1,295	3,479	3,298
Race/Ethnicity	4,684	1,649	2,184	1,295	3,479	3,298
Income	4,506	1,598	2,131	1,267	3,398	3,236
Education in Years	4,680	1,649	2,184	1,294	3,478	3,297
Relationship	4,649	1,637	2,169	1,291	3,460	3,298
Alcohol-Related						
Drinking Every Day Week+	2,745	1,649	2,184	1,295	3,479	3,297
No. Drinks Every Day Week+[a]	460	532	1,157	958	2,115	3,085
Blackouts	2,687	1,649	2,184	1,295	3,479	3,297
Age First Intoxication	3,436	1,609	2,169	1,293	3,462	3,289
Age Regular Drinking	2,956	1,578	2,150	1,288	3,438	3,297
Max Drinks[b]	4,680	1,646	2,184	1,295	3,479	3,293
Sought Help/Treatment	2,957	1,647	2,184	1,295	3,479	3,298
Psychiatric Comorbidity						
MDD	3,015	1,073	1,437	801	2,238	1,757
ASPD	4,621	1,614	2,132	1,237	3,369	3,017
SUD[c]	4,684	1,649	2,184	1,295	3,479	3,298
Theta ERO	1,885	682	953	578	1,531	1,320
Delta ERO	1,885	682	953	578	1,531	1,320
P300 Amplitude	1,696	607	829	521	1,350	1,097
AUD PGS						
AA Subsample	694	175	214	175	389	516
EA Subsample	1,671	688	1,007	582	1,589	1,448

Note: AUD = alcohol use disorder; MDD = major depressive disorder; ASPD = antisocial personality disorder; SUD = comorbid substance use disorder; ERO = event related oscillations; PGS = polygenic score; AA = African-American ancestry; EA = European ancestry

Comparison sample sizes varied across correlates according to patterns of missing data.

[a] Sample sizes for maximum number of drinks consumed every day during period of drinking every day for a week or more are restricted based on endorsement of ever drinking every day for a week or more

[b] Maximized over available interviews

[c] DSM-5 cannabis use disorder, cocaine use disorder, opiate use disorder, simulant use disorder, sedative use disorder, other drug use disorder; DSM-IV nicotine dependence

© 2023 Miller AP et al. *JAMA Network Open.*

eTable 3. Results from mixed effect logistic AUD PGS regression models in COGA cross-sectional sample

**European ancestry subsample (*n* = 5,396)**	**OR**	**95% CI**	***P***
**Criterion severity**			
High-Risk Mild-to-Moderate AUD *vs.* Low-Risk Mild-to-Moderate AUD	1.10	(0.98-1.24)	1.11×10^{-1}
Severe AUD *vs.* Low-Risk Mild-to-Moderate AUD	1.08	(0.98-1.19)	1.27×10^{-1}
Severe AUD *vs.* High-Risk Mild-to-Moderate AUD	0.94	(0.84-1.07)	3.50×10^{-1}
**DSM-5 categories**			
Moderate AUD *vs.* Mild AUD	1.07	(0.96-1.19)	2.04×10^{-1}
Severe AUD *vs.* Mild AUD	1.07	(0.97-1.19)	1.76×10^{-1}
Severe AUD *vs.* Moderate AUD	0.98	(0.88-1.09)	7.26×10^{-1}
AUD *vs.* No AUD	**1.16**	**(1.09-1.24)**	$\mathbf{8.69 \times 10^{-6}}$
Mild-to-moderate *vs.* No AUD	**1.13**	**(1.04-1.21)**	$\mathbf{1.97 \times 10^{-3}}$
Severe AUD *vs.* No AUD	**1.23**	**(1.12-1.35)**	$\mathbf{1.57 \times 10^{-5}}$

**African-American ancestry subsample (*n* = 1,774)**	**OR**	**95% CI**	***P***
**Criterion severity**			
High-Risk Mild-to-Moderate AUD *vs.* Low-Risk Mild-to-Moderate AUD	1.11	(0.91-1.35)	3.25×10^{-1}
Severe AUD *vs.* Low-Risk Mild-to-Moderate AUD	**1.30**	**(1.09-1.56)**	$\mathbf{4.33 \times 10^{-3}}$
Severe AUD *vs.* High-Risk Mild-to-Moderate AUD	1.08	(0.90-1.29)	4.13×10^{-1}
**DSM-5 categories**			
Moderate AUD *vs.* Mild AUD	1.11	(0.91-1.36)	2.99×10^{-1}
Severe AUD *vs.* Mild AUD	**1.27**	**(1.07-1.51)**	$\mathbf{7.02 \times 10^{-3}}$
Severe AUD *vs.* Moderate AUD	1.10	(0.92-1.32)	2.84×10^{-1}
AUD *vs.* No AUD	**1.13**	**(1.01-1.26)**	$\mathbf{3.67 \times 10^{-2}}$
Mild-to-moderate *vs.* No AUD	1.02	(0.89-1.17)	7.24×10^{-1}
Severe AUD *vs.* No AUD	**1.27**	**(1.10-1.47)**	$\mathbf{1.47 \times 10^{-3}}$

Note: AUD = alcohol use disorder; PGS = polygenic score; OR = odds ratio
Fixed covariates: age, age^2, sex, 10 PCs, genotyping array (for European subsample), cohort
Random covariates: family ID
Ancestry grouping based on PC and reported race/ethnicity (Black non-Hispanic, white non-Hispanic)
African-American ancestry sample AUD PGS on AUD criterion count: $\beta = 0.26$, SE = 0.08, $P = 1.04 \times 10^{-3}$
European ancestry sample AUD PGS on AUD criterion count: $\beta = 0.20$, SE = 0.05, $P = 9.36 \times 10^{-6}$

© 2023 Miller AP et al. *JAMA Network Open.*

eTable 4. Alcohol-related, psychiatric comorbidity, electroencephalography, and AUD polygenic score correlate sample sizes for COGA cross-sectional cohort organized by low- and high-risk mild-to-moderate and severe AUD

	Mild-to-Moderate AUD		
	Endorsed Low-Risk Criteria	Endorsed High-Risk Criteria	Severe AUD
Variables	*n* = 2,486	*n* = 993	*n* = 3,298
Alcohol-Related			
Drinking Every Day Week+	2,486	993	3,297
No. Drinks Every Day Week+[a]	1,422	693	3,085
Blackouts	2,486	993	3,297
Age First Intoxication	2,474	988	3,289
Age Regular Drinking	2,459	979	3,297
Max Drinks[b]	2,486	993	3,293
Sought Help/Treatment	2,486	993	3,298
Psychiatric Comorbidity			
MDD	1,627	611	1,757
ASPD	2,437	932	3,017
SUD[c]	2,486	993	3,298
Theta ERO	1,094	437	1,320
Delta ERO	1,094	437	1,320
P300 Amplitude	966	384	1,097
AUD PGS			
AA Subsample	204	185	516
EA Subsample	1,204	385	1,448

Note: AUD = alcohol use disorder; MDD = major depressive disorder; ASPD = antisocial personality disorder; SUD = comorbid substance use disorder; ERO = event related oscillations; PGS = polygenic score; AA = African-American ancestry; EA = European Ancestry

Comparison sample sizes varied across correlates according to patterns of missing data.

[a] Sample sizes for maximum number of drinks consumed every day during period of drinking every day for a week or more are restricted based on endorsement of ever drinking every day for a week or more

[b] Maximized over available interviews

[c] DSM-5 cannabis use disorder, cocaine use disorder, opiate use disorder, simulant use disorder, sedative use disorder, other drug use disorder; DSM-IV nicotine dependence

© 2023 Miller AP et al. *JAMA Network Open.*

eTable 5. Results from mixed linear and logistic regression models comparing alcohol-related, psychiatric, electroencephalography, and polygenic score correlates across low-risk mild-to-moderate, high-risk mild-to-moderate, and severe AUD in COGA cross-sectional cohort

	High-risk mild-to-moderate AUD *vs.* low-risk mild-to-moderate AUD			Severe AUD *vs.* high-risk mild-to-moderate AUD			Severe AUD *vs.* low-risk mild-to-moderate AUD		
Variables	OR/β	95% CI	*P*	OR/β	95% CI	*P*	OR/β	95% CI	*P*
Alcohol-Related									
Drinking Every Day Week+	1.14	(0.93-1.39)	2.00E-01	**4.04**	**(3.12-5.23)**	**2.42E-26**	**4.61**	**(3.45-6.15)**	**3.14E-25**
No. Drinks Every Day Week+	**0.15**	**(0.05-0.25)**	**2.25E-03**	**0.52**	**(0.42-0.61)**	**8.56E-27**	**0.67**	**(0.55-0.78)**	**1.02E-29**
Blackouts	0.96	(0.79-1.15)	6.41E-01	**2.55**	**(2.06-3.15)**	**3.17E-18**	**2.44**	**(1.91-3.11)**	**8.66E-13**
Age First Intoxication	0.08	(0.00-0.15)	5.02E-02	**-0.29**	**(-0.37-0.21)**	**7.20E-12**	**-0.21**	**(-0.31-0.11)**	**2.02E-05**
Age Regular Drinking	0.06	(-0.02-0.13)	1.65E-01	**-0.26**	**(-0.34-0.18)**	**9.77E-10**	**-0.20**	**(-0.30-0.11)**	**4.60E-05**
Max Drinks	**0.08**	**(0.01-0.15)**	**3.49E-02**	**0.58**	**(0.50-0.66)**	**6.24E-47**	**0.66**	**(0.57-0.75)**	**4.66E-44**
Sought Help/Treatment	**1.48**	**(1.19-1.85)**	**5.39E-04**	**7.18**	**(5.86-8.81)**	**4.49E-80**	**10.63**	**(8.24-13.72)**	**1.46E-73**
Psychiatric Comorbidity									
MDD	**1.53**	**(1.12-2.10)**	**7.57E-03**	**2.07**	**(1.45-2.94)**	**5.08E-05**	**3.17**	**(2.07-4.83)**	**9.29E-08**
ASPD	**1.56**	**(1.17-2.08)**	**2.19E-03**	**1.84**	**(1.40-2.42)**	**1.45E-05**	**2.87**	**(2.04-4.04)**	**1.70E-09**
SUD	**1.34**	**(1.09-1.63)**	**4.39E-03**	**2.61**	**(2.08-3.28)**	**1.06E-16**	**3.49**	**(2.70-4.52)**	**2.69E-21**
Theta ERO	**-0.12**	**(-0.24-0.00)**	**4.25E-02**	-0.09	(-0.22-0.04)	1.67E-01	**-0.21**	**(-0.36-0.06)**	**4.88E-03**
Delta ERO	0.00	(-0.12-0.12)	9.66E-01	-0.05	(-0.18-0.08)	4.37E-01	-0.05	(-0.20-0.10)	5.23E-01
P300 Amplitude	-0.08	(-0.20-0.03)	1.61E-01	-0.10	(-0.22-0.03)	1.26E-01	**-0.18**	**(-0.32-0.04)**	**1.36E-02**
AUD PGS									
EA Subsample	0.09	(-0.01-0.20)	8.06E-02	0.02	(-0.09-0.13)	7.39E-01	0.07	(-0.01-0.16)	7.04E-02
AA Subsample	0.12	(-0.28-0.05)	1.63E-01	0.10	(-0.09-0.29)	3.02E-01	**0.22**	**(0.06-0.38)**	**7.35E-03**

Note: AUD = alcohol use disorder; MDD = major depressive disorder; ASPD = antisocial personality disorder; SUD = comorbid substance use disorder; ERO = event related oscillations; PGS = polygenic score; AA = African-American ancestry; EA = European ancestry; OR = odds ratio; β = standardized regression coefficient

© 2023 Miller AP et al. *JAMA Network Open.*

eResults. Supplemental results from survival analyses

In the longitudinal sample (*N*=2,818), a total of 252 individuals eventually met criteria for severe AUD (i.e., 8.9% of total sample; median onset age of 24 years). Cox proportional hazards regression models demonstrated increasing hazards as a function of prior criterion count-based severity: single (*n*=566) *vs.* no criteria (*n*=1,236; adjusted hazard ratio [aHR], 1.13; 95% CI, 0.66-1.93); mild AUD (*n*=833; aHR, 3.48; 95% CI, 2.37-5.11; 14.7% transition); and moderate AUD (*n*=183; aHR, 11.30; 95% CI, 7.33-17.43; 40.4% transition; between mild and moderate AUD aHR, 3.25; 95% CI, 2.10-5.03). In total, 1,016 individuals in the sample (36.1%) met criteria for mild-to-moderate AUD prior their final assessment, and 196 of these individuals (19.3%) went on to develop severe AUD at a subsequent timepoint. Cox proportion hazards analyses similarly found that a prior mild-to-moderate AUD diagnosis was associated with a significantly elevated hazards of severe AUD after adjusting for covariates (aHR, 11.30; 95% CI, 7.33-17.43; Figure 2), though this result was primarily driven by those endorsing high-risk criteria (see main text).

Other youth characteristics that have been previously linked to progression to severe AUD were also significantly associated with progression to severe AUD; however, adjusted hazards for these characteristics ranged from aHR, 0.75-0.79 for age at onset variables (i.e., first drink, regular drinking, and first intoxication; inverse aHR, 1.27-1.33) to aHR=7.88 for other SUDs, suggesting in general that these indices are not as predictive of transitioning to severe AUD as either a prior moderate AUD diagnosis or a high-risk mild-to-moderate AUD diagnosis. In multivariate models including all risk factor variables in addition to covariates mentioned above (i.e., accounting for shared variance among these feature), high-risk mild-to-moderate was the strongest predictor (aHR, 4.25; 95% CI, 2.57-7.04) followed by other SUDs (aHR, 3.65; 95% CI, 2.42-5.52). Similar models for prior mild *vs.* moderate AUD also demonstrated an association between a prior diagnosis of moderate AUD and progression to severe AUD (aHR, 4.46; 95% CI, 2.70-7.35) that was larger in magnitude than other youth characteristics (other SUDs aHR, 3.76; 95% CI, 2.49-5.68).

© 2023 Miller AP et al. *JAMA Network Open.*

eReferences

1. American Psychiatric Association. *Diagnostic and Statistical Manual of Mental Disorders: DSM-IV.* 4th ed. Washington D.C.: 1994.

2. American Psychiatric Association. *Diagnostic and Statistical Manual of Mental Disorders: DSM-5*. 5th ed. Washington D.C.: 2013.

3. Hesselbrock M, Easton C, Bucholz KK, Schuckit M, Hesselbrock V. A validity study of the SSAGA--A comparison with the SCAN. *Addiction.* 1999;94(9):1361-1370. doi:10.1046/j.1360-0443.1999.94913618.x

4. Bucholz KK, Cadoret R, Cloninger CR, et al. A new, semi-structured psychiatric interview for use in genetic linkage studies: A report on the reliability of the SSAGA. *J Stud Alcohol*. 1994;55(2):149-158.

5. Bucholz KK, McCutcheon VV, Agrawal A, et al. Comparison of parent, peer, psychiatric, and cannabis use influences across stages of offspring alcohol involvement: Evidence from the COGA prospective study. *Alcohol Clin Exp Res*. 2017;41(2):359-368. doi:10.1111/acer.13293

6. Johnson EC, Salvatore JE, Lai D, et al. The Collaborative Study on the Genetics of Alcoholism: Genetics. [published online ahead of print, 2023 June 30]. *Genes Brain Behav*. 2023;e12856. doi:10.1111/gbb.12856

7. Zhou H, Sealock JM, Sanchez-Roige S, et al. Genome-wide meta-analysis of problematic alcohol use in 435,563 individuals yields insights into biology and relationships with other traits. *Nat Neurosci*. 2020;23(7):809-818. doi:10.1038/s41593-020-0643-5

8. Walters RK, Polimanti R, Johnson EC, et al. Transancestral GWAS of alcohol dependence reveals common genetic underpinnings with psychiatric disorders. *Nat Neurosci*. 2018;21(12):1656-1669. doi:10.1038/s41593-018-0275-1

9. Kurki MI, Karjalainen J, Palta P, et al. FinnGen provides genetic insights from a well-phenotyped isolated population. *Nature*. 2023;613(7944):508-518. doi:10.1038/s41586-022-05473-8

10. Ge T, Chen CY, Ni Y, Feng YCA, Smoller JW. Polygenic prediction via Bayesian regression and continuous shrinkage priors. *Nat Commun*. 2019;10(1):1776. doi:10.1038/s41467-019-09718-5

11. Ruan Y, Lin YF, Feng YCA, et al. Improving polygenic prediction in ancestrally diverse populations. *Nat Genet*. 2022;54(5):573-580. doi:10.1038/s41588-022-01054-7

12. Kranzler HR, Zhou H, Kember RL, et al. Genome-wide association study of alcohol consumption and use disorder in 274,424 individuals from multiple populations. *Nat Commun*. 2019;10(1):1-11. doi:10.1038/s41467-019-09480-8

13. Chang CC, Chow CC, Tellier LCAM, Vattikuti S, Purcell SM, Lee JJ. Second-generation PLINK: Rising to the challenge of larger and richer datasets. *GigaScience*. 2015;4(1):1-16. doi:10.1186/s13742-015-0047-8

14. Bates D, Mächler M, Bolker B, Walker S. Fitting linear mixed-effects models using lme4. *J Stat Softw*. 2015;67(1):1-48. doi:10.18637/jss.v067.i01

15. Kahler CW, Strong DR. A rasch model analysis of DSM-IV alcohol abuse and dependence items in the National Epidemiological Survey on Alcohol and Related Conditions. *Alcohol Clin Exp Res*. 2006;30(7):1165-1175. doi:10.1111/j.1530-0277.2006.00140.x

16. Saha TD, Chou SP, Grant BF. Toward an alcohol use disorder continuum using item response theory: Results from the National Epidemiologic Survey on Alcohol and Related Conditions. *Psychol Med*. 2006;36(7):931-941. doi:10.1017/S003329170600746X

© 2023 Miller AP et al. *JAMA Network Open.*

17. Embretson SE, Reise SP. *Item Response Theory*. Psychology Press: 2000.

18. Rosseel Y. lavaan: An R package for structural equation modeling. *J Stat Softw*. 2012;48:1-36. doi:10.18637/jss.v048.i02

19. The R Foundation for Statistical Computing. The R Project for Statistical Computing. https://www.r-project.org/. 2023. Accessed May 15, 2023.

20. Boness CL, Lane SP, Sher KJ. Not all alcohol use disorder criteria are equally severe: towards severity grading of individual criteria in college drinkers. *Psychol Addict Behav*. 2019;33(1):35-49. doi:10.1037/adb0000443

© 2023 Miller AP et al. *JAMA Network Open.*
