# Supplementary material for: Diagnostic Criteria for Identifying Individuals at High Risk of Progression From Mild or Moderate to Severe Alcohol Use Disorder
Source: JAMA Netw Open. 2023 Oct 10;6(10):e2337192. doi: 10.1001/jamanetworkopen.2023.37192 (PMC10565602; doi:10.1001/jamanetworkopen.2023.37192)
Supplement: Supplement 2. — Nonauthor Collaborators [file jamanetwopen-e2337192-s002.pdf]

Supplemental Online Content: Nonauthor Collaborators
*First name, last name, and suffix (if applicable) are required and will appear in PubMed.

*Group Name(s): Collaborative Study on the Genetics of Alcoholism (COGA)							
***First Name and Middle Initial(s)**	***Last Name**	***Suffix (eg, Jr, III)**	Academic Degrees	Institution	Location (city, state/province, country)	Role or Contribution, eg, chair, principal investigator	Group (if more than 1 Group listed in the byline) and/or Subgroup (eg, Steering Committee)
Victor	Hesselbrock		PhD	University of Connecticut	Farmington, CT, USA	Principal Investigator	
Tatiana	Foroud		PhD	Indiana University	Indianapolis, IN, USA	Principal Investigator	
Yunlong	Liu		PhD	Indiana University	Indianapolis, IN, USA	Site Lead	
Samuel	Kuperman		MD	University of Iowa	Iowa City, IA, USA	Site Lead	
Ashwini K.	Pandey		PhD	SUNY Downstate Health Sciences University	Brooklyn, NY, USA	Site Lead	
Laura J.	Bierut		MD	Washington University in St. Louis	St. Louis, MO, USA	Site Lead	
John	Rice		PhD	Washington University in St. Louis	St. Louis, MO, USA	Site Lead	
Jay A.	Tischfield		PhD	Rutgers University	Piscataway, NJ, USA	Site Lead	
Ronald P.	Hart		PhD	Rutgers University	Piscataway, NJ, USA	Site Lead	
Laura	Almasy		PhD	The Children's Hospital of Philadelphia; University of Pennsylvania	Philadelphia, PA, USA	Site Lead	
Alison	Goate		Dphil	Icahn School of Medicine at Mount Sinai	New York City, NY, USA	Site Lead	
Paul	Slesinger		PhD	Icahn School of Medicine at Mount Sinai	New York City, NY, USA	Site Lead	
Denise M.	Scott		PhD	Howard University	Washington, DC, USA	Site Lead	
Lance O.	Bauer		PhD	University of Connecticut	Farmington, CT, USA	Collaborator	
John I.	Nurnberger	Jr	MD, PhD	Indiana University	Indianapolis, IN, USA	Collaborator	
Leah	Wetherill		PhD	Indiana University	Indianapolis, IN, USA	Collaborator	
Xiaoling	Xuei		PhD	Indiana University	Indianapolis, IN, USA	Collaborator	
Dongbing	Lai		PhD	Indiana University	Indianapolis, IN, USA	Collaborator	
Sean J.	O'Connor		MD	Indiana University	Indianapolis, IN, USA	Collaborator	
Grace	Chan		PhD	University of Iowa; University of Connecticut	Farmington, CT, USA	Collaborator	
David B.	Chorlian		MS	SUNY Downstate Health Sciences University	Brooklyn, NY, USA	Collaborator	
Jian	Zhang		PhD	SUNY Downstate Health Sciences University	Brooklyn, NY, USA	Collaborator	
Peter B.	Barr		PhD	SUNY Downstate Health Sciences University	Brooklyn, NY, USA	Collaborator	
Gayathri	Pandey		PhD	SUNY Downstate Health Sciences University	Brooklyn, NY, USA	Collaborator	
Niamh	Mullins		PhD	Icahn School of Medicine at Mount Sinai	New York City, NY, USA	Collaborator	
Andrey P.	Anokhin		PhD	Washington University in St. Louis	St. Louis, MO, USA	Collaborator	
Sarah	Hartz		MD, PhD	Washington University in St. Louis	St. Louis, MO, USA	Collaborator	
Scott	Saccone		PhD	Washington University in St. Louis	St. Louis, MO, USA	Collaborator	
Jennifer C.	Moore		PhD	Rutgers University	Piscataway, NJ, USA	Collaborator	
Fazil	Aliev		PhD	Rutgers University	Piscataway, NJ, USA	Collaborator	
Zhiping	Pang		MD, PhD	Rutgers University	Piscataway, NJ, USA	Collaborator	
Alison	Merikangas		MPH, PhD	The Children's Hospital of Philadelphia; University of Pennsylvania	Philadelphia, PA, USA	Collaborator	
Hemin	Chin		PhD	National Institute of Alcohol Abuse and Alcoholism	Bethesda, MD, USA	Staff Collaborator	
Abbas	Parsian		PhD	National Institute of Alcohol Abuse and Alcoholism	Bethesda, MD, USA	Staff Collaborator	
