# Supplementary material for: Diagnostic Criteria for Identifying Individuals at High Risk of Progression From Mild or Moderate to Severe Alcohol Use Disorder
Source: JAMA Netw Open. 2023 Oct 10;6(10):e2337192. doi: 10.1001/jamanetworkopen.2023.37192 (PMC10565602; doi:10.1001/jamanetworkopen.2023.37192)
Supplement: Supplement 3. — Data Sharing Statement [file jamanetwopen-e2337192-s003.pdf]

Data Sharing Statement

Miller. Diagnostic Criteria for Identifying Individuals at High Risk of Progression From Mild or Moderate to Severe Alcohol Use Disorder. *JAMA Netw Open*. Published October 10, 2023. doi:10.1001/jamanetworkopen.2023.37192

Data
Data available: No

Additional Information
Explanation for why data not available: COGA data are available through the National Institute on Alcohol Abuse and Alcoholism or the database of Genotypes and Phenotypes (dbGaP; phs000763.v1.p1, phs000125.v1.p1). PGC alcohol dependence GWAS summary statistics may be obtained from the PGC website (https://www.med.unc.edu/pgc/). MVP GWAS summary statistics are available through dbGaP (phs001672). FinnGenR8 ICD-based AUD GWAS data were obtained from https://r8.finngen.fi/pheno/AUD. For more information, visit https://finngen.gitbook.io/documentation/.
